# Comprehensive analysis of adverse events associated with vortioxetine using the FDA adverse event reporting system

**DOI:** 10.3389/fphar.2025.1519865

**Published:** 2025-05-02

**Authors:** Liangxia Li, Qianqian Xu, Liangfang Pang, Yarui Liu, Yuanyuan Lu

**Affiliations:** Department of Pharmacy, Maternal and Child Health Hospital of Hubei Province, Tongji Medical College, Huazhong University of Science and Technology, Wuhan, China

**Keywords:** vortioxetine, adverse events, FAERS, disproportionality analysis, safety

## Abstract

**Background:**

Vortioxetine is a novel antidepressant belonging to the class of selective serotonin reuptake inhibitors. This study aims to comprehensively analyze the adverse events (AEs) associated with vortioxetine by analyzing the FDA Adverse Event Reporting System (FAERS) database.

**Methods:**

This study collected reports of vortioxetine as primary suspected drug in FAERS database from the fourth quarter of 2013 to the fourth quarter of 2023. We conducted disproportionality analysis to quantify signals of AEs using the Reporting Odds Ratio (ROR), Proportional Reporting Ratio (PRR), Bayesian Confidence Propagation Neural Network (BCPNN) and Multi-item Gamma-Poisson Shrinker (MGPS).

**Results:**

A total of 12,279 reports of vortioxetine as the primary suspected drug and 30,104 AEs were identified. 51.57% of AE reports originated from consumers and 45.85% from health professional. The AEs associated with vortioxetine involved 27 different system organs (SOCs). A total of 158 AE signals of vortioxetine were identified, including some common adverse events such as nausea, vomiting, and unexpected AE signals such as vision blurred, bruxism, disturbance in attention, akathisia, restless legs syndrome, urinary retention, and electrocardiogram QT prolonged. Gender-specific analysis showed high-risk AEs were different for females (nausea, vomiting, crying, contusion, weight increased, pruritus) and males (completed suicide, negative thoughts, anorgasmia, libido decreased, urinary retention, sexual dysfunction). The median onset time of AEs was 7 days (interquartile range [IQR] 0–30 days), and most AEs (75.10%) occurred within the first month after initiation of vortioxetine.

**Conclusion:**

Our study identified potential new AE signals, offering a broader understanding of the safety profile of vortioxetine, and providing valuable references for its clinical monitoring and further research. It should be noted that nearly half of the reports originated from patients, highlighting the value of patient-reported data in pharmacovigilance, but also reminding us of the need for cautious interpretation due to potential self-reporting biases.

## 1 Introduction

Major depressive disorder (MDD) is a common mental disorder that affects approximately 185 million people worldwide (Marx et al., 2023). The symptoms of MDD include depressed mood, reduced interest or pleasure in previously enjoyable activities, and recurrent thoughts of death (Marx et al., 2023). MDD is characterized by recurrent episodes, imposing a heavy burden on individuals, families and society (Marwaha et al., 2023). In the United States, the economic burden of adults with MDD has significantly increased over time, rising from $236 billion in 2010 to $326 billion in 2018 (Greenberg et al., 2021). Agents that target monoamine neurotransmission (such as serotonin, noradrenaline, and dopamine) are the mainstay of drug treatment for MDD (Greenberg et al., 2021; Njenga et al., 2024). However, due to the multifaceted etiology of MDD, research has uncovered that only approximately one-third of patients achieve remission with the first antidepressant treatment (Mills et al., 2021), and treatment resistance is common (Njenga et al., 2024).

Vortioxetine is a novel antidepressant, approved by FDA in September 2013 for the treatment of adult patients with MDD (Tritschler et al., 2014). Compared with other drugs for MDD, vortioxetine has a unique mechanism of action. It acts as an inhibitor of the serotonin (5-HT) transporter, 5-HT_1A_ receptor agonist, 5-HT_1B_ receptor partial agonist, 5-HT_3_, 5-HT_7_, and 5-HT_1D_ receptor antagonist (Mills et al., 2021). Vortioxetine has a long half-life of approximately 57 h, which is thought to partly explain its low rate of discontinuation symptoms (DS) (Sanchez et al., 2015). Siwek et al. reported that 8 out of 263 patients (3%) who had taken vortioxetine developed discontinuation syndrome DS after drug withdrawal. Among the group of patients experiencing DS, vortioxetine was more frequently discontinued abruptly and without prior medical consultation (Siwek et al., 2021). Clinical trials have demonstrated the efficacy of vortioxetine in the treatment of MDD as well as in the prevention of relapse (Mills et al., 2021; Thase et al., 2022). Some studies have consistently confirmed the efficacy of vortioxetine in improving anhedonia in MDD patients (Cao et al., 2019; McIntyre et al., 2021; Serretti, 2025), as well as in treating elderly depression and bipolar disorder (Dai et al., 2025; Rodrigues Alessi et al., 2025). Vortioxetine is a first-line antidepressant recommended by Canadian Network for Mood and Anxiety Treatments (CANMAT) (Lam et al., 2024). Furthermore, vortioxetine has been recommended by National Institute for Health and Care Excellence (NICE) as an option when there has been no or limited response to at least two antidepressants (NICE, 2022). Vortioxetine has also been observed to improve the cognitive symptoms associated with MDD. The recent network meta-analysis has shown that vortioxetine is the only antidepressant that improves cognitive dysfunction compared to placebo in the digit symbol substitution test (Baune et al., 2018). In recent years, there has been an increasing number of studies on the clinical application of vortioxetine. There is a study documenting the use of vortioxetine in the treatment of nine patients with depressive symptoms accompanied by epilepsy, with all patients achieving relief from their depressive symptoms (Siwek et al., 2022). A pooled analysis of 13 randomized, placebo-controlled trials demonstrated that vortioxetine was also effective in the treatment of patients with MDD accompanied by common comorbid somatic diseases (Baldwin et al., 2022). Some studies have indicated that vortioxetine has the potential to become a therapeutic option for pain syndromes, such as burning mouth syndrome and neuropathic pain (Adamo et al., 2021a; Adamo et al., 2021b; Eliaçık and Erdogan Kaya, 2024; Adamo et al., 2025).

Although vortioxetine exhibits clear therapeutic effects and advantages, its safety remains an important aspect requiring continuous evaluation. In clinical studies, the most common adverse events (AEs) related to vortioxetine include nausea, vomiting, dry mouth, headache, and dizziness (Baldwin et al., 2016; Findling et al., 2022). Given the relatively small sample size and short observation period of clinical trials, the AEs reported in these trials cannot fully capture the safety profile of vortioxetine in a clinical setting. Consequently, it is essential to conduct comprehensive and large-scale monitoring studies. The FDA Adverse Event Reporting System (FAERS) boasts a vast database of AE reports, serving as an essential resource for post-marketing surveillance and early detection of drug safety issues (Jiang et al., 2024). In this study, we analyzed potential AEs of vortioxetine from the FAERS, and evaluated the potential correlation between vortioxetine and AEs through disproportionality analysis. The purpose of this study is to comprehensively investigate the safety of vortioxetine, providing valuable insights for clinical use and further research.

## 2 Methods

### 2.1 Data source

The data for this study are procured from the FAERS database, which undergoes a quarterly update process. Each quarterly FAERS data package comprises several distinct subsets: Patient Demographic Information (DEMO), Drug Information (DRUG), Adverse events (REAC), Patient outcomes (OUTC), Report Source information (RPSR), Drug Therapy Start and End Date (THER), and Drug indication information (INDI). Furthermore, starting from the first quarter of 2019, a new subset named DELETED is introduced, encompassing withdrawn or recalled reports. We have downloaded all reports spanning from the fourth quarter of 2013 to the fourth quarter of 2023.

### 2.2 Data extraction and analysis

Following the FDA’s guidance, we employed MySQL 8.0 to interlink these subsets and eliminate duplicate reports. The deduplication process entailed selecting the latest report based on the CASEID and FDA_DT fields (Ying et al., 2024). If the CASEID (the number used to identify a report) was the same, we selected the report with the latest FDA_DT (the date of the report being received by FDA). If both CASEID and FDA_DT were identical, the report with the higher PRIMARYID (the unique identifier for the report) was chosen. After data deduplication, we removed the withdrawn or recalled reports based on the CASEID listed in the DELETED subset. In order to ensure comprehensive coverage, the search was performed using both the generic name (vortioxetine) and the brand names (brintellix, trintellix). The role_code of AEs includes primary suspected (PS), secondary suspect (SS), concomitant (C), and interaction (I) (Cheng et al., 2024). To enhance the accuracy of the results, we selected the role_code as “PS” in the DRUG files. The Medical Dictionary for Regulatory Activities (MedDRA) serves as a standardized medical terminology that facilitates the reporting and analysis of AE data. Its terminology structure is organized into five hierarchical levels: lowest level terms (LLTs), preferred terms (PTs), high level terms (HLTs), high level group terms (HLGTs), and system organ classes (SOCs) (Brown, 2004). In our study, the AEs were coded using the PTs and then mapped to their corresponding SOC level in MedDRA (version 26.1).

Disproportionality analysis including reporting odds ratio (ROR), proportional reporting ratio (PRR), Bayesian confidence propagation neural network (BCPNN) and multi-item gamma-Poisson shrinker (MGPS) were used to identify the potential positive signals (Jiang et al., 2024). ROR and PRR, as non-Bayesian methods, may exhibit better performance in early signal detection, whereas the Bayesian approach (including BCPNN and MGPS) showcases a strong detection power for unique signals even when there are few AEs reported for the drug. In order to capitalize on their individual strengths and improve the comprehensiveness of signal detection, this research integrates these four disparate analysis methods, consequently expanding the range of signal detection (Cui et al., 2023). The four algorithms are based on a two-by-two contingency table (Table 1). The specific formulas and criteria for these algorithms are shown in Table 2. To ensure accurate detection, a positive signal was generated when all four algorithmic criteria were simultaneously met. Beyond the threshold, a larger value signifies a stronger signal strength. We categorized the positive signals that were not listed in the package insert as “unexpected signals”. In order to identify the gender differences in AEs of vortioxetine as the primary suspected drug, the obtained positive signals were further analyzed by a modified ROR method (Supplementary Table S1) (Zou et al., 2024). We calculated the *p* value through the chi-square test and subsequently adjusted it with the application of the false discovery rate (FDR) method. If the ROR exceeds 1 and the *p* value adjusted by FDR (P.adj) falls below 0.05, it implies a higher likelihood for females to report a specific AE compared to males. Conversely, when the ROR is below 1 and P.adj is less than 0.05, it suggests that males have a greater tendency to report the specified AE than females. To visually describe the the gender differences in the potential AEs of vortioxetine, we crafted a volcano plot where the vertical axis showcased the value of -Log_10_(P.adj), and the horizontal axis displayed the Log_2_(ROR) value. Furthermore, after the exclusion of inaccurate or incomplete date inputs, we assessed the time to onset (TTO) of potential AEs of vortioxetine. The TTO was determined as the period between EVENT_DT (the date on which the AE occurred) and START_DT (the date of vortioxetine initiation). All statistical analyses were executed using R version 4.3.2, while data visualization was accomplished through Origin software (version 2022).

**TABLE 1:** A two-by-two contingency table.

Drug category	Target adverse event	Other adverse events	Total
Vortioxetine	a	b	a + b
Other drugs	c	d	c + d
Total	a + c	b + d	a + b + c + d

a: the number of reports that contain both the target drug and the target adverse event; b: the number of reports that contain the target drug with other adverse events; c: the number of reports that contain the target adverse event related to other drugs; d: the number of reports that contain other drugs and other adverse events.

**TABLE 2:** Overview of the algorithms used for disproportionality analysis.

Algorithms	Formulas	Criteria
ROR	$ROR=\frac{ad}{bc}$	lower limit of 95% CI > 1; *N* ≥ 3
	$95\%CI=e^{In\left(ROR\right)\pm 1.96\sqrt{\frac{1}{a}+\frac{1}{b}+\frac{1}{c}+\frac{1}{d}}}$	
PRR	$PRR=\frac{a/\left(a+b\right)}{c/\left(c+d\right)}$	PRR > 2; χ^2^ ≥ 4; N ≥ 3
	$\chi^{2}=\frac{{\left(ad-bc\right)}^{2}\left(a+b+c+d\right)}{\left(a+b\right)\left(c+d\right)\left(a+c\right)\left(b+d\right)}$	
BCPNN	$IC=\log_{2}\frac{a\left(a+b+c+d\right)}{\left(a+b\right)\left(a+c\right)}$	IC_025_ > 0
	$IC_{025}=E\left(IC\right)-2\sqrt{V\left(IC\right)}$	
MGPS	$EBGM=\frac{a\left(a+b+c+d\right)}{\left(a+c\right)\left(a+b\right)}$	EBGM_05_ > 2
	$95\%CI=e^{In\left(EBGM\right)\pm 1.96\sqrt{\frac{1}{a}+\frac{1}{b}+\frac{1}{c}+\frac{1}{d}}}$	

95% CI: 95% confidence interval; N: the number of reports; χ^2^: chi-squared; ROR: reporting odds ratio; PRR: proportional reporting ratio; BCPNN: bayesian confidence propagation neutral network; MGPS: multi-item gamma Poisson shrinker; IC: information component; IC_025_: the lower limit of the 95% CI, of the IC; E (IC): the IC, expectations; V (IC): the variance of IC; EBGM: empirical Bayesian geometric mean; EBGM_05_: the lower limit of the 95% CI, of EBGM.

## 3 Results

### 3.1 Basic characteristics of AE reports

**FIGURE 1:**
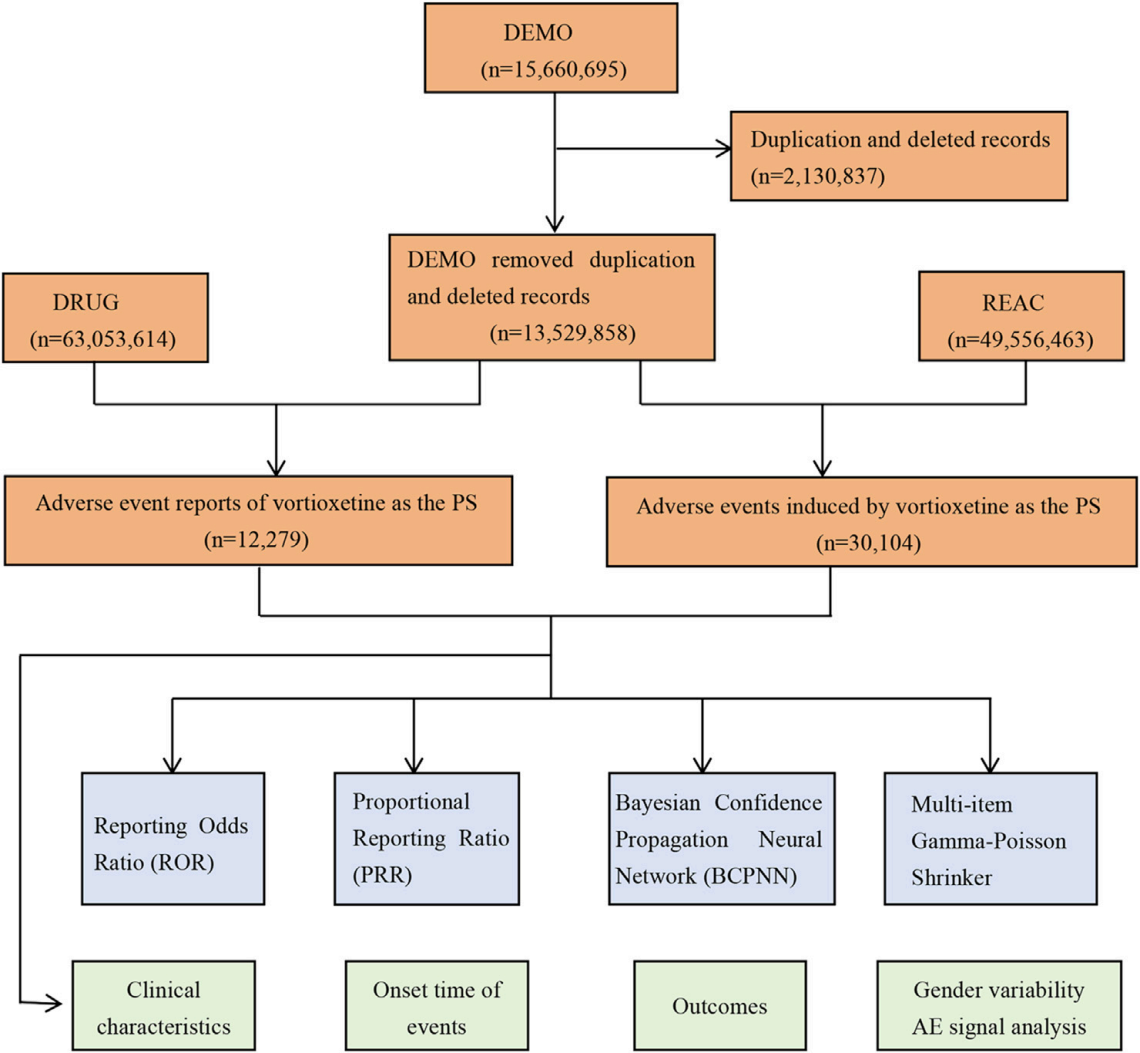
The process of selecting potential adverse events (AEs) of vortioxetine from FDA adverse event reporting database.

During the study period spanning from the fourth quarter of 2013 to the fourth quarter of 2023, the FAERS database received a total of 15,660,695 reports. The detailed process and results of data extraction are shown in Figure 1. After filtering out duplicate and deleted reports, we selected 12,279 reports that identified vortioxetine as primary suspected drug, corresponding to 30,104 AEs. Table 3 presents a summary of the basic characteristics of the reports associated with vortioxetine. Among these reports, the proportion of females (61.20%) surpassed that of males (26.57%). Patients were predominantly aged between 18 and 64 years old in the reports containing age information. Among the 2,461 reports with weight information, the group 50–100 kg made up the largest proportion. The majority of reports originated from the United States (78.52%), followed by Japan (3.81%), and France (2.57%). In terms of the source of the reports, consumers were the main source (51.57%), followed by physicians (26.13%). Regarding the reported outcomes, hospitalization, death, disability, life-threatening events were reported in 1,410 (11.48%), 354 (2.88%), 206 (1.68%) and 195 (1.59%) reports, respectively.

**TABLE 3:** The characteristics of the reports of vortioxetine as the primary suspected drug.

Characteristics	Number of reports	Proportion (%)
Number of reports	12,279	
Gender		
Female	7,515	61.20
Male	3,263	26.57
Unknown	1,501	12.22
Age (year)		
<18	130	1.06
18–64	4,885	39.78
>64	1,155	9.41
Unknown	6,109	49.75
Weight (kg)		
<50	144	1.17
50–100	1,979	16.12
>100	338	2.75
Unknown	9,818	79.96
Reporter Country		
United States	9,641	78.52
Japan	468	3.81
France	315	2.57
Canada	239	1.95
Great Britain	170	1.38
other	1,446	11.78
Reported person		
Consumer (CN)	6,332	51.57
Physician(MD)	3,209	26.13
Other health professional (OT)	1,350	10.99
Health professional (HP)	791	6.44
Pharmacist(PH)	281	2.29
Laywer (LW)	4	0.03
Unknown	312	2.54
Serious outcome		
Other serious important medical event (OT)	3,505	28.54
Hospitalization - initial or prolonged (HO)	1,410	11.48
Death (DE)	354	2.88
Disability (DS)	206	1.68
Life-threatening (LT)	195	1.59
Required intervention to prevent permanent impairment/damage (RI)	11	0.09
Congenital anomaly (CA)	16	0.13

### 3.2 Signal detection at the SOC level

AEs associated with vortioxetine were distributed among 27 SOCs. The top three SOCs ranked by the number of reports were psychiatric disorders (n = 6,851), gastrointestinal disorders (n = 4,614), and nervous system disorders (n = 3,993). The detailed information pertaining to the signal strength at the SOC level is presented in Table 4. The significant SOCs that at least one of the four algorithms met the criteria were psychiatric disorders (ROR: 5.14, 95%CI: 5.00–5.28), gastrointestinal disorders (ROR: 1.98, 95%CI: 1.92–2.05), nervous system disorders (ROR: 1.76, 95%CI: 1.71–1.82), skin and subcutaneous tissue disorders (ROR: 1.14, 95%CI: 1.09–1.19), surgical and medical procedures (ROR: 1.17, 95%CI: 1.07–1.27), reproductive system and breast disorders (ROR: 1.58, 95%CI: 1.43–1.74), ear and labyrinth disorders (ROR: 1.35, 95%CI: 1.16–1.56), and social circumstances (ROR: 1.30, 95%CI: 1.12–1.51). Notably, psychiatric disorders were the only SOC that simultaneously met the criteria of the four algorithms.

**TABLE 4:** Signal strength of potential adverse events of vortioxetine at the System Organ Class (SOC) level.

SOC	Number of reports	ROR (95% CI)	PRR (χ^2^)	IC (IC_025_)	EBGM (EBGM_05_)
Psychiatric disorders	6,851	5.14 (5.00–5.28)	4.20 (17575.80)	2.07 (2.03)	4.19 (4.07)
Gastrointestinal disorders	4,614	1.98 (1.92–2.05)	1.83 (1905.61)	0.87 (0.83)	1.83 (1.78)
Nervous system disorders	3,993	1.76 (1.71–1.82)	1.66 (1144.02)	0.73 (0.68)	1.66 (1.61)
General disorders and administration site conditions	3,982	0.70 (0.67–0.72)	0.74 (461.43)	−0.44 (−0.49)	0.74 (0.71)
Injury, poisoning and procedural complications	2,290	0.65 (0.62–0.68)	0.68 (402.18)	−0.57 (−0.63)	0.68 (0.65)
Skin and subcutaneous tissue disorders	1,899	1.14 (1.09–1.19)	1.13 (30.18)	0.18 (0.11)	1.13 (1.08)
Investigations	1,256	0.71 (0.67–0.75)	0.72 (144.50)	−0.47 (−0.56)	0.72 (0.68)
Musculoskeletal and connective tissue disorders	672	0.42 (0.39–0.45)	0.43 (534.89)	−1.22 (−1.33)	0.43 (0.40)
Eye disorders	604	1.03 (0.95–1.12)	1.03 (0.52)	0.04 (−0.08)	1.03 (0.95)
Metabolism and nutrition disorders	556	0.89 (0.82–0.97)	0.89 (7.46)	−0.17 (−0.29)	0.89 (0.82)
Surgical and medical procedures	484	1.17 (1.07–1.27)	1.16 (11.15)	0.22 (0.08)	1.16 (1.06)
Respiratory, thoracic and mediastinal disorders	431	0.30 (0.27–0.33)	0.31 (688.60)	−1.68 (−1.82)	0.31 (0.28)
Cardiac disorders	401	0.59 (0.53–0.65)	0.59 (115.07)	−0.76 (−0.90)	0.59 (0.54)
Reproductive system and breast disorders	394	1.58 (1.43–1.74)	1.57 (81.76)	0.65 (0.50)	1.57 (1.42)
Infections and infestations	287	0.17 (0.15–0.19)	0.18 (1141.73)	−2.48 (−2.65)	0.18 (0.16)
Vascular disorders	268	0.44 (0.39–0.50)	0.45 (188.55)	−1.17 (−1.34)	0.45 (0.40)
Renal and urinary disorders	218	0.37 (0.32–0.42)	0.37 (231.60)	−1.41 (−1.61)	0.38 (0.33)
Ear and labyrinth disorders	177	1.35 (1.16–1.56)	1.34 (15.57)	0.43 (0.21)	1.34 (1.16)
Social circumstances	171	1.30 (1.12–1.51)	1.30 (11.99)	0.38 (0.16)	1.30 (1.12)
Immune system disorders	131	0.37 (0.31–0.44)	0.37 (140.07)	−1.42 (−1.67)	0.37 (0.31)
Neoplasms benign, malignant and unspecified (incl cysts and polyps)	102	0.11 (0.09–0.14)	0.11 (716.61)	−3.12 (−3.39)	0.11 (0.09)
Hepatobiliary disorders	99	0.40 (0.33–0.49)	0.40 (88.05)	−1.31 (−1.59)	0.40 (0.33)
Blood and lymphatic system disorders	83	0.17 (0.14–0.21)	0.17 (340.58)	−2.55 (−2.85)	0.17 (0.14)
Endocrine disorders	71	0.93 (0.74–1.17)	0.93 (0.37)	−0.10 (−0.44)	0.93 (0.74)
Pregnancy, puerperium and perinatal conditions	24	0.20 (0.13–0.30)	0.20 (76.07)	−2.31 (−2.84)	0.20 (0.14)
Congenital, familial and genetic disorders	23	0.27 (0.18–0.41)	0.27 (44.16)	−1.86 (−2.41)	0.27 (0.18)
Product issues	23	0.04 (0.03–0.07)	0.04 (486.13)	−4.51 (−5.04)	0.04 (0.03)

95% CI: 95% confidence interval; χ^2^: chi-squared; ROR: reporting odds ratio; PRR: proportional reporting ratio; IC: information component; IC_025_: the lower limit of the 95% CI, of the IC; EBGM: empirical Bayesian geometric mean; EBGM_05_: the lower limit of the 95% CI, of EBGM.

### 3.3 Signal detection at the PT level

**FIGURE 2:**
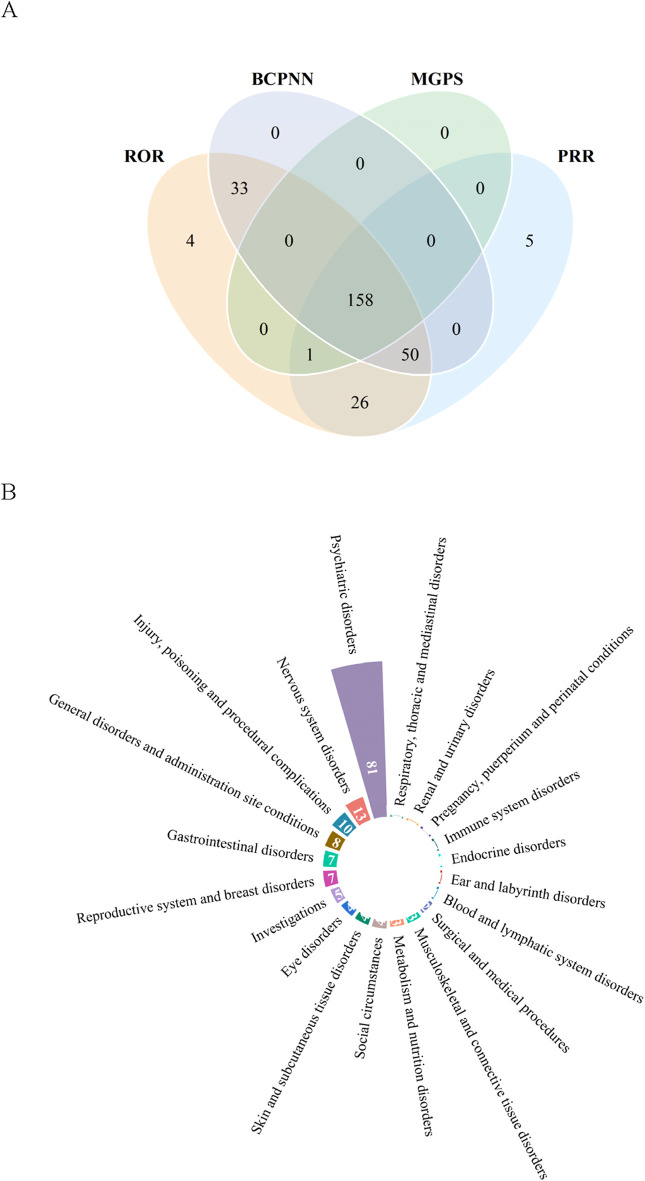
Signals detection at the preferred term (PT) level. **(A)** Venn diagram of PT signals that met the criteria of four algorithms. **(B)** The System Organ Class (SOC) attribution of the 158 PTs that simultaneously satisfied the criteria of the four algorithms.

Upon integration of the criteria from all the algorithms, we ultimately identified 158 positive signals at the PT level. Figure 2A showcases a venn diagram, visually depicting the PT signals that comply with the criteria of the four algorithms. The PT signals were distributed across 20 SOCs, and we arranged these SOCs in descending order based on the number of PT signals (Figure 2B). The signal strength of PTs with a count greater than 100 is presented in Table 5, encompassing 35 PTs and 10 corresponding SOCs, and the remaining PT signals are shown in Supplementary Table S2. In our study, PTs such as nausea (n = 1,885), suicidal ideation (n = 783), anxiety (n = 740), vomiting (n = 707), feeling abnormal (n = 583), pruritus (n = 562), insomnia (n = 554), asthenia (n = 471), weight increased (n = 461), irritability (n = 416), anger (n = 390), constipation (n = 298) were presented, which were consistent with the AEs described in the package insert. Notably, our disproportionality analysis identified many unexpected and significant AE signals which were not previously mentioned in the package insert, including disturbance in attention (n = 390), apathy (n = 288), vision blurred (n = 170), panic attack (n = 96), eating disorder (n = 84), akathisia (n = 57), electrocardiogram QT prolonged (n = 56), urinary retention (n = 56), restless legs syndrome (n = 46), and bruxism (n = 40). In addition, the AEs mentioned in the package insert, including headache (n = 633), dizziness (n = 486), diarrhoea (n = 409), failed to satisfy the criteria of at least one of the four algorithms.

**TABLE 5:** Signal strength of AEs with a count >100 at the Preferred Term (PT) level.

SOC	PT	Number of reports	ROR (95%CI)	PRR (χ^2^)	IC (IC_025_)	EBGM (EBGM_05_)
Eye disorders	Vision blurred*	170	2.73 (2.35–3.18)	2.72 (185.57)	1.44 (1.21)	2.72 (2.34)
Gastrointestinal disorders	Nausea	1,885	5.29 (5.05–5.55)	5.03 (6132.05)	2.32 (2.25)	5.01 (4.78)
Gastrointestinal disorders	Vomiting	707	3.35 (3.11–3.61)	3.30 (1136.25)	1.72 (1.60)	3.29 (3.05)
Gastrointestinal disorders	Constipation	298	2.86 (2.55–3.21)	2.85 (357.10)	1.51 (1.33)	2.84 (2.53)
Gastrointestinal disorders	Dry mouth	130	3.50 (2.95–4.16)	3.49 (230.51)	1.80 (1.52)	3.48 (2.93)
General disorders and administration site conditions	Feeling abnormal	583	4.82 (4.44–5.23)	4.75 (1725.11)	2.24 (2.11)	4.73 (4.36)
General disorders and administration site conditions	Asthenia	471	2.66 (2.43–2.91)	2.63 (479.32)	1.40 (1.26)	2.63 (2.40)
General disorders and administration site conditions	Crying*	122	7.53 (6.30–9.00)	7.50 (684.19)	2.90 (2.57)	7.47 (6.25)
Injury, poisoning and procedural complications	Contusion*	122	2.60 (2.18–3.11)	2.60 (119.79)	1.38 (1.10)	2.59 (2.17)
Investigations	Weight increased	461	4.44 (4.05–4.87)	4.39 (1205.67)	2.13 (1.98)	4.38 (3.99)
Metabolism and nutrition disorders	Hyperphagia*	164	106.73 (90.98–125.19)	106.15 (15793.31)	6.62 (5.72)	98.21 (83.73)
Nervous system disorders	Disturbance in attention*	390	15.31 (13.85–16.93)	15.13 (5090.43)	3.90 (3.71)	14.96 (13.53)
Nervous system disorders	Hypersomnia	215	15.86 (13.85–18.15)	15.75 (2935.49)	3.96 (3.67)	15.57 (13.61)
Nervous system disorders	Tremor	179	2.37 (2.04–2.74)	2.36 (140.14)	1.24 (1.01)	2.36 (2.03)
Nervous system disorders	Serotonin syndrome	121	14.80 (12.37–17.72)	14.75 (1533.80)	3.87 (3.45)	14.59 (12.20)
Psychiatric disorders	Suicidal ideation	783	20.97 (19.52–22.52)	20.45 (14276.36)	4.33 (4.19)	20.15 (18.76)
Psychiatric disorders	Anxiety	740	5.54 (5.15–5.96)	5.43 (2672.34)	2.43 (2.32)	5.41 (5.03)
Psychiatric disorders	Insomnia	554	4.44 (4.09–4.83)	4.38 (1446.80)	2.13 (1.99)	4.37 (4.02)
Psychiatric disorders	Irritability	416	15.12 (13.72–16.67)	14.93 (5349.34)	3.88 (3.69)	14.77 (13.40)
Psychiatric disorders	Anger	390	26.44 (23.91–29.25)	26.11 (9238.72)	4.68 (4.44)	25.62 (23.16)
Psychiatric disorders	Apathy*	288	43.63 (38.78–49.09)	43.22 (11499.24)	5.39 (5.02)	41.86 (37.21)
Psychiatric disorders	Agitation	244	7.76 (6.84–8.81)	7.71 (1416.82)	2.94 (2.71)	7.67 (6.76)
Psychiatric disorders	Mood swings	211	14.79 (12.90–16.94)	14.69 (2663.19)	3.86 (3.57)	14.54 (12.69)
Psychiatric disorders	Libido decreased	197	36.17 (31.38–41.69)	35.94 (6512.92)	5.13 (4.69)	35.00 (30.37)
Psychiatric disorders	Suicide attempt	179	7.07 (6.10–8.20)	7.04 (922.75)	2.81 (2.54)	7.00 (6.04)
Psychiatric disorders	Completed suicide	167	4.72 (4.05–5.49)	4.70 (484.70)	2.23 (1.97)	4.68 (4.02)
Psychiatric disorders	Feeling guilty*	165	277.16 (234.21–327.98)	275.64 (37252.24)	7.83 (6.34)	227.59 (192.32)
Psychiatric disorders	Mania	126	19.05 (15.97–22.72)	18.97 (2114.66)	4.23 (3.78)	18.71 (15.69)
Psychiatric disorders	Abnormal dreams	108	10.57 (8.75–12.78)	10.54 (925.26)	3.39 (2.99)	10.46 (8.65)
Psychiatric disorders	Nightmare	108	7.01 (5.80–8.48)	6.99 (551.81)	2.80 (2.44)	6.96 (5.76)
Reproductive system and breast disorders	Sexual dysfunction	113	21.56 (17.89–25.97)	21.48 (2171.13)	4.40 (3.89)	21.15 (17.55)
Skin and subcutaneous tissue disorders	Pruritus	562	3.32 (3.05–3.61)	3.28 (891.11)	1.71 (1.58)	3.27 (3.01)
Skin and subcutaneous tissue disorders	Hyperhidrosis	227	3.79 (3.33–4.32)	3.77 (462.20)	1.91 (1.70)	3.76 (3.30)
Skin and subcutaneous tissue disorders	Pruritus generalised	150	14.82 (12.61–17.41)	14.75 (1901.30)	3.87 (3.51)	14.59 (12.42)
Surgical and medical procedures	Therapy interrupted	104	3.56 (2.93–4.31)	3.55 (190.03)	1.82 (1.51)	3.54 (2.92)

SOC: system organ class; 95% CI: 95% confidence interval; χ^2^: chi-squared; ROR: reporting odds ratio; PRR: proportional reporting ratio; IC: information component; IC_025_: the lower limit of the 95% CI, of the IC; EBGM: empirical Bayesian geometric mean; EBGM_05_: the lower limit of the 95% CI, of EBGM; Asterisks (*) indicate unexpected signals that are not listed on the package insert.

### 3.4 Gender difference analysis

**FIGURE 3:**
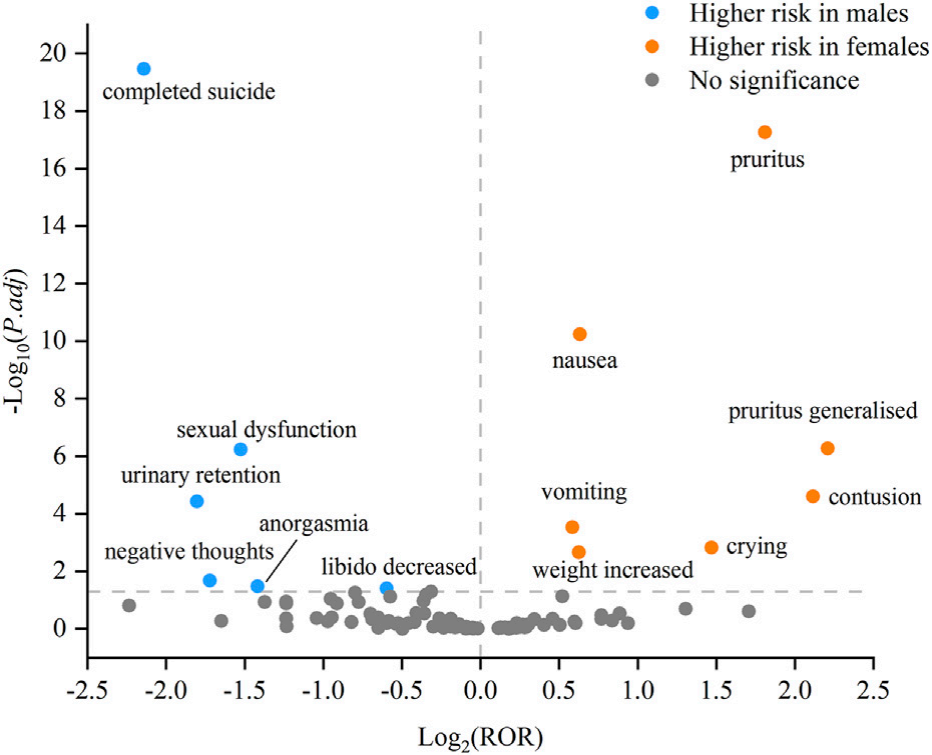
Volcano plot of gender differences in the potential AE signals of vortioxetine.

In order to investigate potential gender differences among the above 158 AE signals, we conducted a statistical analysis by selecting 102 PTs that provided gender information. Based on the modified ROR method, we identified 13 PTs that exhibited gender differences. The specific results are shown in Supplementary Table S3. The higher risk of AEs in females included nausea, vomiting, crying, contusion, weight increased, pruritus, and pruritus generalised. While males had a greater likelihood of experiencing AEs such as completed suicide, negative thoughts, anorgasmia, libido decreased, urinary retention, and sexual dysfunction. To provide a clearer depiction of gender differences, we utilized a volcano plot as a visual tool to highlight significant signals (Figure 3). Each dot within this plot signified a PT of vortioxetine, with orange dots representing the high-risk AE signals in females and blue dots representing those in males.

### 3.5 Time to onset of adverse events

**FIGURE 4:**
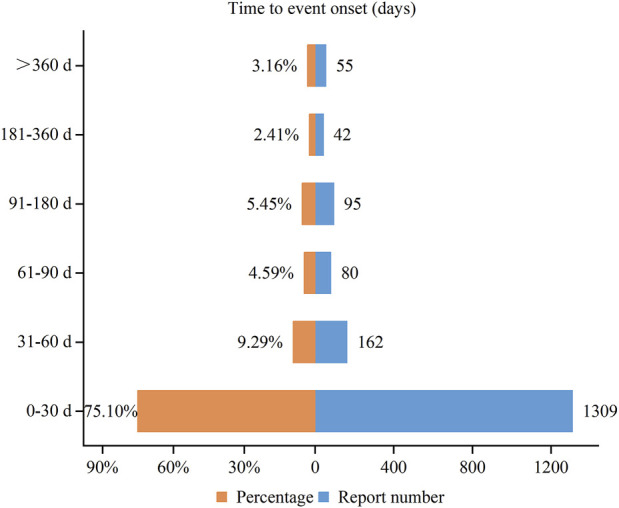
Time to onset of adverse events associated with vortioxetine.

We collected the onset time of potential AEs of vortioxetine and obtained 1,743 reports that provided the onset time of AEs. The median onset time was 7 days (interquartile range [IQR]: 0–30 days). Figure 4 intuitively depicts the specific onset time and its proportion. Most AEs happened within the first month after the administration of vortioxetine, accounting for 75.10%. The incidence rate of AEs exhibited a downward trend over time, with the rates for the second and third months being 9.29% and 4.59%, respectively.

## 4 Discussion

As vortioxetine is increasingly widely used in clinical practice, it is necessary to continuously monitor its safety. In this study, we utilized disproportionality analysis to identify potential AE signals associated with vortioxetine in the FAERS database, enabling the identification of AEs that have not yet been recorded in the package insert. Among the AEs of vortioxetine as the primary suspected drug, it was observed to be significantly higher among females (61.20%) compared to males (26.57%). This may be attributed to the higher prevalence of MDD among females than males (Marx et al., 2023), which subsequently leads to an increased opportunity for drug use. Among the reported age data, the majority of patients were within the age range of 18–64 years, which aligned with the epidemiological characteristics of MDD (Malhi and Mann, 2018). In addition, the small proportion of patients under 18 years old was associated with the fact that the safety and efficacy of vortioxetine in children under 18 years old were not well established. Serious outcomes encompassed death, disability, hospitalization, and life-threatening events. With the increasing concern about drug safety, patients are strongly encouraged to directly submit their AEs to the national pharmacovigilance authority, which can help to avoid underreporting and enables the collection of more detailed descriptions of AEs (Inácio et al., 2017). The AEs in our study were primarily derived from consumer self-reporting, accounting for 51.57%. Since vortioxetine is an oral medication typically self-administered at home, patients are often the first to recognize and report AEs, thereby accounting for the preponderance of consumer reports in our study. Most of the AEs originated from the United States, which may be related to factors such as earlier market availability of vortioxetine in the United States, a larger user base for the drug and a stronger willingness among users to report AEs.

The adverse events associated with vortioxetine, primarily concentrated in psychiatric disorders, gastrointestinal disorders, and nervous system disorders, were in concordance with the safety information presented in the package insert and previous studies (Baldwin et al., 2016; Findling et al., 2022; Wu et al., 2025). It was noteworthy that psychiatric disorders had the highest number of reports and were identified as significant signals through disproportionality analysis. Our study also identified the AEs specified in the drug’s package insert, such as nausea, suicidal ideation, anxiety, vomiting, feeling abnormal, pruritus, insomnia, asthenia, weight increased, irritability, anger, and constipation, demonstrating the credibility of our research methodology.

Among the AE signals of the psychiatric disorders, the top five most frequently reported AEs were suicidal ideation, anxiety, insomnia, irritability, and anger. It is well known that 5-HT is involved in the regulation of sleep, memory, attention, emotion, appetite, and anxiety (Pourhamzeh et al., 2022). These signals may reflect vortioxetine’s modulation of the serotonin system. MDD is a severe disease that itself is a strong predictor of suicide ideation. Antidepressants are crucial in the treatment of MDD, yet the debate persists regarding their potential association with an elevated risk of suicide ideation in some patients. The FDA mandated in 2004 that all antidepressants should carry warnings on increased suicidality risks for children and adolescents diagnosed with MDD, and expanded it in 2007 to cover young adults up to 24 years old (Friedman and Leon, 2007). A study analyzing data from 13 clinical trials found no increased risk of suicidal ideation and behavior in adult MDD patients treated with vortioxetine (Mahableshwarkar et al., 2020). Our study revealed that suicidal ideation was the most commonly reported AEs within the SOC of psychiatric disorders, suggesting the importance of monitoring the risks of suicidal ideation during the treatment of MDD patients with vortioxetine. In addition, some PTs that were not specified in the package insert were identified, such as apathy, feeling guilty, panic attack, eating disorder, and bruxism. Feeling guilty (n = 165) had the highest signal strength, with a ROR of 277.16 (95% CI: 234.21–327.98), a PRR of 275.64 (χ^2^ = 37252.24), an IC of 7.83 (IC_025_ = 6.34), and an EBGM of 227.59 (EBGM_05_ = 192.32). Study has found that guilt is closely related to suicidal ideation (Feiten et al., 2021). Although suicidal ideation, feeling guilty and apathy were detected to be strong positive signals, they may not necessarily represent an adverse reaction to the medication. Instead, these symptoms might simply be part of the clinical presentation of depression and dynamically evolve until significant therapeutic effects are achieved and depressive symptoms are relieved. A post-marketing study using the world pharmacovigilance database has demonstrated an association between bruxism and vortioxetine, which is consistent with our findings (Revet et al., 2020). Bruxism has been related to disturbances in the central dopaminergic system (Bhattacharjee et al., 2022). On the one hand, vortioxetine can enhance the dopamine levels in certain regions of the brain (Gibb and Deeks, 2014). On the other hand, it may induce an elevation in the level of 5-HT (Gibb and Deeks, 2014), which in turn exerts an inhibitory effect on dopamine release. Therefore, it is necessary to conduct further research to clarify the specific role of the dopaminergic system in bruxism of vortioxetine, and to explore whether there are other mechanisms.

Regarding the nervous system, disturbance in attention was not recorded in the package insert, and there were no relevant clinical research reports. Hypersomnia and tremor have been reported in clinical study, with the incidence to be 2.6%–3.2% and 0.3%–1.3% respectively. Serotonin syndrome is a potentially life-threatening outcome associated with excessive serotonergic activity within both the peripheral and central nervous systems (Scotton et al., 2019). One case reported the serotonin syndrom in a 69-year-old female was related to the administration of vortioxetine, which was characterized by hypertension, rigidity in the upper extremities accompanied by intermittent tremors, brisk reflexes throughout the limbs, clonus in the limbs with upgoing plantar responses, and an elevated level of creatine kinase (Ong and Vasanwala, 2018). In addition, headache (n = 633) and dizziness (n = 486) were reported in relatively high numbers in our study and had also shown a high incidence rate in clinical trials (Mahableshwarkar et al., 2015; Baldwin et al., 2016). However, after conducting disproportionality analysis, we did not identify positive signals for these AEs. This could potentially be explained by the fact that, compared to vortioxetine, these AEs were also common among other drugs in the FAERS database, thereby influencing the signal values. The absence of signals did not imply the absence of relative AEs but rather signified that these AEs were not disproportionately common.

Sexual dysfunction is a common adverse reaction of antidepressants, which seriously affects the quality of life and medication compliance of patients (Montejo et al., 2015). We identified several AE signals related to sexual dysfunction, including libido decreased, erectile dysfunction, anorgasmia, loss of libido, orgasm abnormal, ejaculation delayed, and ejaculation failure. In a pooled analysis of seven studies, the incidence of sexual dysfunction were 25.7%–46.1% for vortioxetine 5–20 mg, with only 2.2% of these cases being spontaneously reported by patients (Jacobsen et al., 2016). Contrary to many other AEs, sexual dysfunction is seldom spontaneously reported (De Boer and Schoevers, 2017). Thus, physicians should remain vigilant and attentive to this aspect.

The most reported AE signals among gastrointestinal disorders included nausea, vomiting, constipation, and dry mouth, which aligned with the findings reported in clinical studies (Mahableshwarkar et al., 2015; Baldwin et al., 2016). A study reported that at the recommended therapeutic dose of 5–20 mg per day, the incidence rates of nausea, vomiting, constipation, and dry mouth were 20.9%–31.2%, 2.9%–6.5%, 3.3%–5.6% and 5.7%–7.0%, respectively (Baldwin et al., 2016). In terms of skin and subcutaneous tissue disorders, pruritus and hyperhidrosis were identified as positive signals, with the reported incidence rates being 6.6% for pruritus and 0.5%–2.4% for hyperhidrosis (Baldwin et al., 2016; Adair et al., 2024). The results of our analysis suggested that the potential AE signals of vortioxetine may also occurred in other organs or tissues. Notably, we identified unexpected significant signals including vision blurred, electrocardiogram QT prolonged, akathisia, dyskinesia, restless legs syndrome, and urinary retention. Among these, QT interval prolongation can potentially lead to fatal arrhythmias (Del Rosario et al., 2010). While one study suggested that once-daily vortioxetine (10 and 40 mg) for 14 days did not significantly affect the QT interval in healthy individuals (Wang et al., 2013), given the relatively short duration of this study, further validation was deemed necessary. Restless legs syndrome is characterized by an irresistible urge to move the legs, often accompanied by uncomfortable sensations in the legs, mainly occurring in the evening and at night, and disappearing with movement (Allen et al., 2014). A 54-year-old male reported unpleasant sensations in his legs and an urge to move them at night after 2–3 weeks of vortioxetine use, with significant improvement observed a week after discontinuing vortioxetine (Romigi et al., 2019). The causal relationship between vortioxetine and unexpected AEs is unclear, necessitating further clinical research to elucidate both the causality and the potential mechanisms.

Taking into account gender disparities in assessing drug safety enhances precise management of AEs. Our study provided gender-specific AE profiles. Specifically, we found that females were more likely to develop nausea, vomiting, crying, contusion, weight increased, pruritus, and pruritus generalised. While males were more prone to experience completed suicide, negative thoughts, anorgasmia, libido decreased, urinary retention, and sexual dysfunction. Baldwin et al. found that there was a dose effect of vortioxetine-related nausea and vomiting (Baldwin et al., 2016). The pharmacokinetic study of multiple dosing with vortioxetine showed that females had 27% higher AUC_0-24_ (area under the plasma concentration-time curve from time 0–24 h) and 24% higher C_max_ (maximum plasma concentration) values compared to males (Chen et al., 2018). These findings indicate that females have a higher systemic exposure to vortioxetine after multiple doses, which may lead to a higher risk of nausea and vomiting. However, in addition to the biological factors associated with gender, gendered social factors play a predominant role in leading to this gender difference. Globally, males are more likely to commit suicide than females (Sher, 2020). It has been proposed that factors such as a lack of help-seeking behavior, work stress, impulsivity, alcohol and drug abuse, and the use of highly lethal means can contribute to male suicide (Sher, 2020). Compared with females, males tend to wait for symptoms to subside without intervention, which may lead to a higher likelihood of more serious adverse events in males (Zou et al., 2024). Despite requiring further validation, these findings offer a better reference for drug monitoring in both male and female patients to a certain extent.

A study found that vortioxetine-related nausea symptoms mainly occurred in the first 2 weeks after administration (Baldwin et al., 2016). Our analysis of TTO revealed that the median time for the potential AEs of vortioxetine was 7 days, with most AEs occurring within the first month following vortioxetine treatment (n = 1,309, 75.10%). Furthermore, we noticed a decreasing trend in the incidence of AEs over time. However, it is noteworthy that the TTO of the potential AEs of vortioxetine was missing from most of the reports in our study, which may limit the accurate reflection of the actual onset time. Therefore, patients and clinicians need to be vigilant about the onset time, actively identify adverse events, and promptly implement effective measures.

There are several inherent limitations worth discussing. First, the FAERS database is a spontaneous reporting system, which may lead to reporting biases, including underreporting, inaccurate reporting, and selective reporting. More than half of the reports in our research stemmed from consumers, potentially leading to a bias towards subjective or more dramatic incidents. Second, the absence of total number of patients treated with vortioxetine makes it impossible to calculate the incidence rate of each AE. Third, due to the lack of detailed clinical information, it difficult to control for confounding factors such as dose, treatment duration, concomitant medications, and comorbidities. For example, suicidal ideation may reflect the natural progression of underlying MDD rather than being directly attributable to vortioxetine use, thereby limiting the ability to establish a clear causal relationship between vortioxetine and AEs. Disproportionality analysis can only provide statistical associations rather than definitive causal links. In spite of these limitations, the FAERS database remains a valuable resource for identifying potential drug safety concerns. Our data offer a comprehensive list of case numbers and potential AE signal values of vortioxetine, helping the accumulation of knowledge on the safety profile of vortioxetine.

## 5 Conclusion

We conducted an assessment of the safety characteristics of vortioxetine, utilizing the AE reports submitted to the FAERS database spanning from the fourth quarter of 2013 to the fourth quarter of 2023. According to the disproportionality analysis results, potential AE signals of vortioxetine occurred in multiple organs and tissues, including psychiatric, nervous, gastrointestinal, cutaneous, ocular and reproductive system. The comprehensive and systematic analysis of the FAERS database has indeed identified some unexpected and significant potential AE signals of vortioxetine. However, due to most of the reports came from consumers and the inherent limitations of the FAERS database, it is essential to conduct prospective clinical trials to further validate these findings.

## Data Availability

The original contributions presented in the study are included in the article/Supplementary Material, further inquiries can be directed to the corresponding author.
