# Supplementary material for: Comprehensive analysis of adverse events associated with vortioxetine using the FDA adverse event reporting system
Source: Front Pharmacol. 2025 May 2;16:1519865. doi: 10.3389/fphar.2025.1519865 (PMC12081438; doi:10.3389/fphar.2025.1519865)
Supplement: Supplementary file 3 [file Table1.docx]

**SUPPLEMENTARY TABLE S1**

A two-by-two contingency table for disproportionality analysis of the gender difference.

|  | Target adverse event | Other adverse events | Total |
| --- | --- | --- | --- |
| Feamles | a | b | a+b |
| Males | c | d | c+d |
| Total | a+c | b+d | a+b+c+d |

a: number of reports of target adverse event with vortioxetine in females; b: number of reports of other adverse events with vortioxetine in females; c: number of reports of target adverse event with vortioxetine in males; d: number of reports of other adverse events with vortioxetine in males.

Note: ROR= (a/c)/(b/d); 95%CI=exp (ln(ROR)±1.96*sqrt(1/a +1/b + 1/c + 1/d)).
